# A pulmonary infection by *Actinomyces odontolyticus* and *Veillonella atypica* in an immunocompetent patient with dental caries

**DOI:** 10.1002/rcr2.493

**Published:** 2019-09-30

**Authors:** Ernesto Crisafulli, Nicol Bernardinello, Veronica Alfieri, Francesca Pellegrino, Chiara Lazzari, Letizia Gnetti, Alfredo Chetta

**Affiliations:** ^1^ Department of Medicine and Surgery University of Parma Parma Italy; ^2^ Department of Morphology, Surgery and Experimental Medicine, Intensive Care Unit Sant'Anna Hospital Ferrara Italy; ^3^ Unit of Surgical Pathology University Hospital of Parma Parma Italy

**Keywords:** Pulmonary infection, *Actinomyces odontolyticus*, *Veillonella atypica*, bronchoscopy, dental caries

## Abstract

Actinomycosis is a rare, chronic granulomatous infection, frequently associated with immunocompromised states, but it can also affect healthy people. Here, we report a case of a pulmonary infection by *Actinomyces odontolitycus* and *Veillonella atypica* due to a dental caries in an immunocompetent 65‐year‐old man patient.

## Introduction

Actinomyces species are predominantly anaerobic, non‐motile, gram‐positive bacteria commonly found in human mucosae, particularly in the oral cavity and gastrointestinal and urogenital tracts 1. *Actinomyces majerii*, *Actinomyces odontolyticus*, and particularly *Actinomyces israelii* and *Actinomyces graevenitzii* 1 are the most prevalent pathogens associated with pulmonary infections. Although, in most cases, the infection of the bronchial tree comes directly from the oral cavity, the dissemination may be haematogenous, especially in immunocompromised patients 1. In general, Actinomyces infections are cause of dental caries and periodontitis, but less frequently, they can lead to peritonitis or brain or lung infections with abscess 2.

## Case Report

A 65‐year‐old male was admitted in June 2016 to our Lung Unit with a history of productive cough and intermittent fever in the last month and dyspnoea on exertion in the last week. No haemoptysis or chest pain was reported. He was a farmer and an active smoker of 35 pack‐years. His medical history was negative for previous relevant conditions; thus, he was not on any medication. He denied drug abuse or allergies. Poor oral hygiene and an irregular diet with a frequent consumption of alcohol with meals (one or two glasses of wine) were reported by the patient. At admission, cyanosis and clubbing were absent. Blood pressure was 120/80 mmHg, pulse was 97 beats/min, respiratory rate was 21 breaths/min, temperature was 37.2°C, and oxygen peripheral saturation was 96% in room air. Physical examination showed absence of lung sound in the upper and medium area of the right haemithorax. Initial laboratory exams were remarkable for a white cell count of 15.4·10^3^/μL with a predominant neutrophil count of 89% and for a C‐reactive protein (CRP) of 158.2 mg/L.

**Figure 1:**
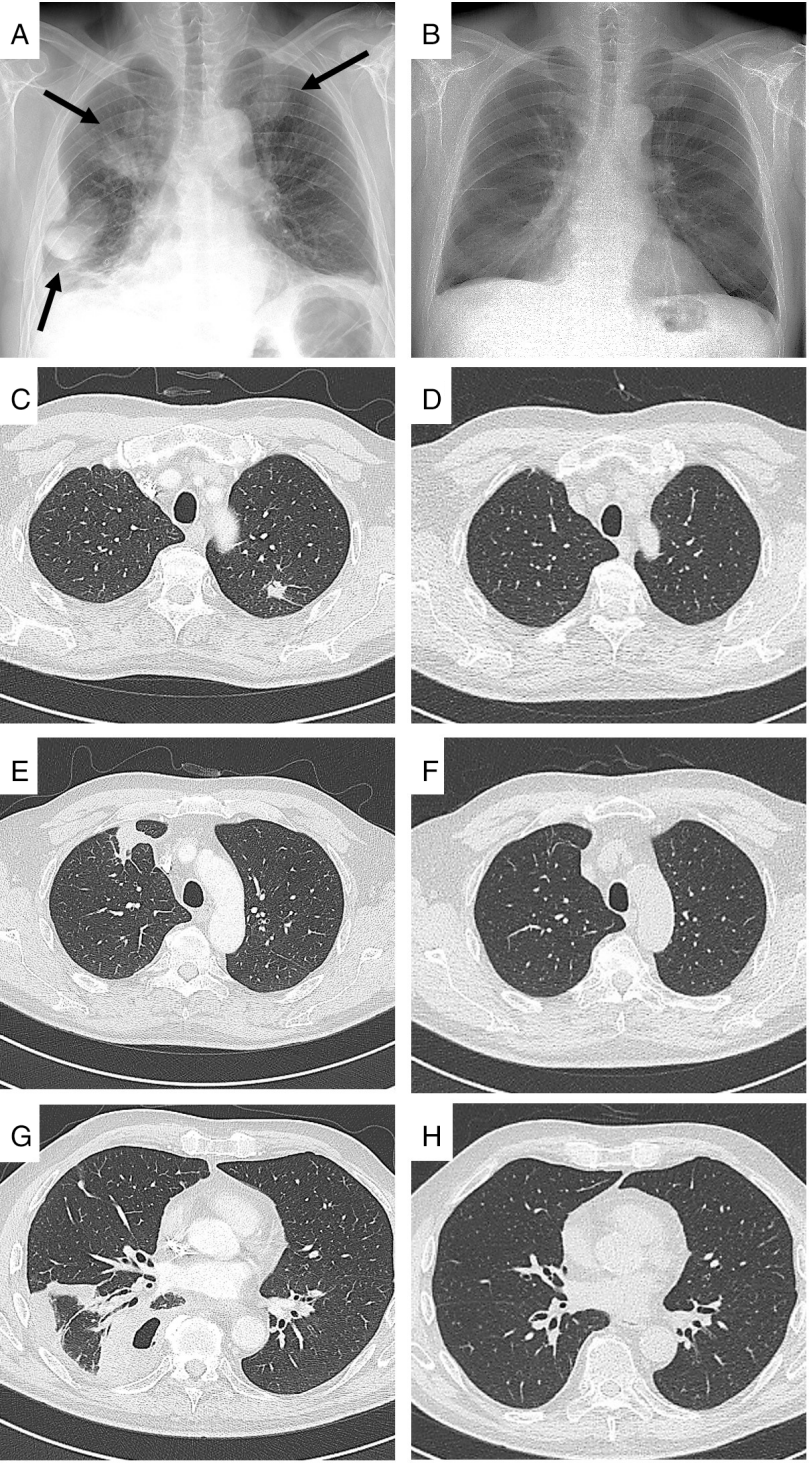
Chest X‐ray (A) and computed tomography (CT) scan (C, E, G) at admission. Chest X‐ray at discharge (B). CT scan at three months' follow‐up (D, F, H).

**Figure 2:**
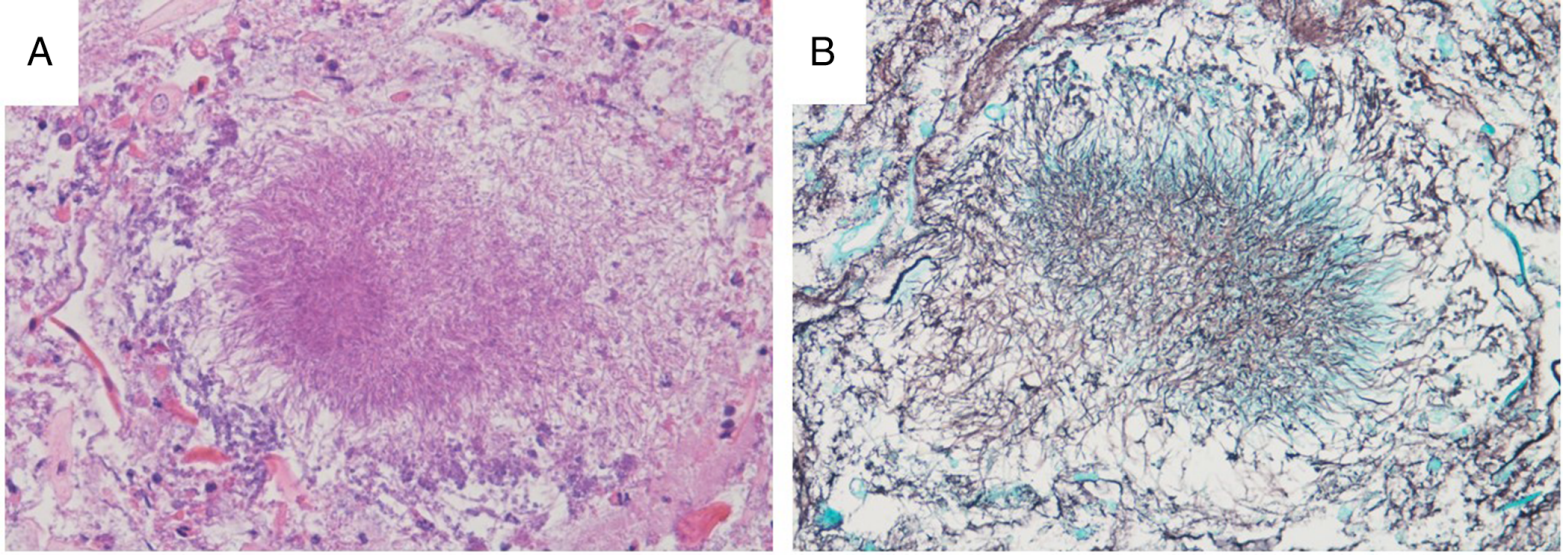
Periodic Acid Schiff (A) and Grocott (B) colourations showing the fungal hyphae.

Chest X‐ray (Fig. 1A, black arrows) and a consecutive chest computed tomography (CT) scan (Fig. 1C, E, G) demonstrated an organized pleural effusion on the right side associated with a basal lesion containing air inside and two dense consolidations in the left and right superior lobes. A chest CT‐guided transthoracic fine‐needle aspiration cytology (FNAC) of the right basal lesion was performed. During this procedure, due to the presence of purulent liquid, we also performed a pleural aspiration, in which we removed 230 cc of dense and purulent liquid rich in neutrophils. Cultures of this pleural liquid were negative for pathogens. A flexible fibreoptic bronchoscopy was also performed. Cultures of the sample obtained by bronchial aspirate showed colonies of *A. odontolyticus* and *Veillonella atypica*. Matrix‐assisted laser desorption/ionization time‐of‐flight mass spectrometry for the identification of pathogens was performed. The Periodic Acid Schiff (PAS) (Fig. 2A) and Grocott (Fig. 2B) colourations identify the fungal hyphae in a cytological sample of bronchial aspirate. Transbronchial biopsy samples demonstrated purulent non‐specific inflammation, and no neoplastic cells were observed. Culture for *Mycobacterium tuberculosis* was negative. The orthopantomogram indicated severe periodontal disease with diffuse dental caries.

The patient was started on empiric antibiotic therapy with metronidazole (1500 mg/day) and piperacillin‐tazobactam (13.5 g/day) for a duration of three weeks. His clinical condition improved significantly, CRP dropped to 3.15 mg/L, and the chest X‐ray showed (Fig. 1B) reduction in the pulmonary lesions. He was discharged home with metronidazole (1500 mg/day) for a further 14 days. On follow up at three months, CT showed complete resolution (Fig. 1D, F, H).

## Discussion

Due to the rarity of the infection and its various clinical manifestations, pulmonary actinomycosis remains a challenging diagnosis. It may in fact mimic other lung disease, such as pulmonary tuberculosis or lung cancer 3. In this case, our first suspicion was lung cancer, and thus, we first performed a transthoracic FNAC that, however, showed a purulent infection. Consequently, we performed a bronchoscopy that was useful in the diagnosis of actinomycosis infection. The origin was confirmed by the orthopantomogram, describing diffuse dental caries and cause of the infection. The chronic dental caries and poor oral hygiene may have favoured oral traumatism, mucosal abrasion, and microorganism proliferations, and the pulmonary actinomycosis likely resulted from the aspiration of oropharyngeal secretions into the respiratory tract. Actinomyces bacteria may destroy the mucosa, disseminating and invading other tissues. Factors that facilitate this process are related to cervico‐facial and dental surgery (in case of localization in the mouth) or abdominal traumas, pelvic infections, and the use of intrauterine or intravaginal devices.

Thoracic actinomycosis represents around 15–20% of Actinomyces infections 4, and the presence of synergistic bacteria in actinomycosis is not rare, involving bacteria such as *Bacteroides*, *Fusobacterium*, or *Streptococcus* spp. 1. In this context, the presence of the *V. atypica* in our patient has probably played a role as co‐infection. Poor oral hygiene, alcohol consumption, malnutrition, chronic respiratory disease, and especially immunocompromised states can promote pulmonary infection by actinomycosis. In immunocompromised patients, severe lung infections may spread to close tissues (e.g. pericardium, ribs, or thoracic skin), potentially disseminating the infection to peripheral muscles and subcutaneous tissue. Currently, there are no guidelines in the literature for the treatment; however, the recommended therapy for actinomyces infections is two to six weeks of intravenous penicillin followed by 6–12 months of oral therapy 5. To our knowledge, the case we reported is the first in Italy concerning a pulmonary infection by *A. odontolyticus* and *V. atypica* in an immunocompetent patient with dental caries as the direct cause of infection. The collection of an infected sample by bronchoscopy was crucial for the diagnosis.

### Disclosure Statement

Appropriate written informed consent was obtained for publication of this case report and accompanying images.
